# Deciphering Viral Replication Dynamics in Feline Infectious Peritonitis: A Quantitative Approach

**DOI:** 10.3390/v17020279

**Published:** 2025-02-18

**Authors:** Carole J. Burgener, Emi N. Barker, Teodoru Soare, Diana-Gabriela Soare, Andrea M. Spiri, Alexandra J. Malbon, Marina L. Meli, Anja Kipar

**Affiliations:** 1 Institute of Veterinary Pathology, Vetsuisse Faculty, University of Zurich (IVPZ), Winterthurerstrasse 268, 8057 Zürich, Switzerland; carole.burgener@uzh.ch; 2 Center for Clinical Studies, Vetsuisse Faculty, University of Zurich, Winterthurerstrasse 260, 8057 Zurich, Switzerland; aspiri@vetlabor.ch (A.M.S.); mmeli@vetclinics.uzh.ch (M.L.M.); 3 Langford Vets, University of Bristol, Langford BS40 5DU, UK; emi.barker@bristol.ac.uk; 4 Bristol Vet School, University of Bristol, Langford BS40 5DU, UK; 5 Department of Pathology, Faculty of Veterinary Medicine, University of Agronomic Sciences and Veterinary Medicine of Bucharest, Splaiul Independentei Street, No. 105, Sector 5, 050097 Bucharest, Romania; teodoru.soare@gmail.com; 6 Histovet, Str. Sirenelor 81-83, Sector 5, 50855 Bucharest, Romania; soare.dianag@gmail.com; 7 Clinical Laboratory, Department of Clinical Diagnostics and Services, Vetsuisse Faculty, University of Zurich, Winterthurerstrasse 260, 8057 Zurich, Switzerland; 8 The Royal (Dick) School of Veterinary Studies, Easter Bush Campus, University of Edinburgh, Edinburgh EH8 9YL, UK; alexandra.malbon@ed.ac.uk

**Keywords:** FCoV, FIP, RT-qPCR, viral genomic and messenger RNA, envelope protein, RNA-dependent RNA polymerase

## Abstract

Feline infectious peritonitis (FIP) is a complex immune-mediated disease caused by feline coronavirus (FCoV). Despite advancements in understanding its pathogenesis, challenges persist in elucidating viral factors related to virion composition and replication. This study examined FCoV-infected cats with and without FIP for potential associations with or variation in expression ratios for different viral genes. We analyzed tissue samples with FIP lesions from 46 cats with FIP and mesenteric lymph nodes from 10 FCoV-infected cats (7b RT-qPCR positive) without FIP with three RT-qPCR assays, targeting (sub)-genomic RNAs of the RNA-dependent RNA polymerase (RdRp) and envelope (E) genes. In cats with FIP, the RdRp mRNA assay yielded the highest copy numbers, followed by the combined RdRp gRNA and mRNA assay; the E mRNA assay yielded the lowest copy numbers. In cats without FIP, significantly fewer viral RNA copies were detected regardless of the assay. Viral gene expression was not detected in six and was observed only at low levels in one or more assays in four samples. The observed correlation between assays and the intragroup correlation assay indicate consistent transcription of both the structural E protein and RdRp genes within FIP lesions in cats with FIP but not in cats with systemic FCoV infection alone.

## 1. Introduction

Feline infectious peritonitis (FIP) is caused by feline coronavirus (FCoV), an alphacoronavirus and member of the family *Coronaviridae* within the order *Nidovirales*. FCoV is an enveloped, positive-stranded, linear RNA virus [1]. It is widely accepted that it is transmitted feco-orally, resulting in an enteric infection, often with no, or only mild and transient, clinical signs [2]. Interestingly, while the prevalence of FCoV infection is high, especially in multi-cat households [3,4,5], FIP only manifests in a small proportion of infected animals [6,7,8]. In the recent past, a lot of emphasis was placed on identifying viral mutations that are directly linked to the pathogenicity of FCoV, especially focusing on amino acid variations in the spike (S) protein [9,10]. It has been suggested that differences in cleavage site positioning and activation mechanisms between different FCoVs could contribute to variations in pathogenicity [11]. Supporting this hypothesis, in 2013, a study found that a change in the viral genome encoding an amino acid substitution (methionine to leucine at position 1058, M1058L) in the fusion peptide of S was associated with increased virulence, which allowed for the differentiation of FIP lesion-associated FCoVs from FCoVs in the feces of cats without FIP in 92% of cases [9]. A later publication showed that the viral genomic mutation was present in non-enteric tissue-associated FCoVs from cats with and without FIP, suggesting that this mutation was associated with systemic FCoV spread rather than being specific to FIP [12,13]. Another study failed to confirm the presence of S gene mutations in the tissues of nine FCoV-infected animals without FIP that had been detected using a commercial RT-qPCR [14]. Additional studies have shown that for cats with FIP, detection of S gene mutations is dependent upon tissue type [15]. In addition to viral factors, multiple studies have suggested a hereditary predisposition of the host to disease, and particularly linked variants of genes associated with immune response to viral infection [16,17,18].

FIP is an immunopathological disease [19,20]. Recent studies have highlighted that differences in Toll-like receptors (TLRs) and inflammatory pathways, e.g., increased levels of inflammatory cytokines such as IL-1β and TNF-α, are involved in the pathogenesis of FIP, shedding light on the complex interactions between the virus and the host’s immune response that influence disease onset and progression [21]. A transcriptomic analysis of mesenteric lymph nodes (MLNs) showed distinct differences in gene expression between FCoV-infected cats with and without FIP. While viral infection generally triggered an increase in antiviral responses, this effect was more prominent in cats with FIP. Cats with FIP also exhibited heightened pro-inflammatory activity in the MLN, whereas FCoV-infected cats without FIP showed less intense humoral immune activation (lower expression levels of genes involved in immunoglobulin production and B cell activation). Notably, immune processes related to T cells were suppressed in FIP cases, highlighting the role of immune imbalance in the progression of the disease [22].

During coronavirus replication and transcription, genomic and subgenomic RNAs are produced, with negative-strand intermediates [23]. Firstly, a full-length negative-sense genomic copy is prepared, which serves as the template for the synthesis of the positive-sense genomic RNA [24]. For the production of subgenomic mRNAs, discontinuous viral transcription, a characteristic feature of most viruses in the order Nidovirales, is initiated [25]. This implies that the replication and transcription complex stops transcription after the recognition of so-called transcription regulatory sequences upstream of most open reading frames (ORFs), at the 3′ third of the genome [24]. Each subgenomic mRNA encompasses a 5′ leader sequence that mirrors the genomic 5′ end, serving as a conserved starting point for positive-strand RNA synthesis [26]. The RNA-dependent RNA polymerase (RdRp) is responsible for the transcription and replication of the viral RNA, ensuring the production of new virions. As a key determinant of coronaviral replication fidelity and efficiency, variations within the RdRp gene can profoundly impact viral fitness, pathogenicity, and response to antiviral interventions [27,28].

FCoV virions are composed of four structural proteins, the spike (S), envelope (E), membrane (M), and nucleocapsid (N) proteins, encoded at the 3′ end of the viral genome [23]. The S protein, a large glycoprotein present as peplomers projecting from the surface of virions, facilitates receptor binding and gives the viral particle the characteristic “solar corona” appearance [23,29]. The M protein has essential functions in viral assembly and morphogenesis. It plays a crucial role in shaping the virion’s structure and facilitating viral budding and release from infected cells [23]. The M protein is also involved in interactions with other viral and host cell components, contributing to virus–host interactions and pathogenesis. For instance, virus-like particles (VLPs) form when coronaviral M protein is co-expressed with the E protein, indicating that these two proteins interact to promote the production of the viral envelope [30]. However, the specific protein requirements for VLP release vary between coronavirus species [31]. Currently, there are no published data available regarding the specific requirements for VLP formation of FCoV. One study mentioned that only the M and E proteins are required but did not provide data to directly support this hypothesis [32]. The smaller E protein, an 8–12 kDa membrane protein, is a so-called “viroporin” with ion channel activity [33]. Although the E protein is present in the viral envelope in smaller quantities than the S and M proteins [34], it plays a crucial role in virus assembly and maintaining the stability of the viral envelope [35,36,37,38]. By interacting with other viral structural proteins, the E protein facilitates the formation of viral particles in infected cells [32]. The E protein is also implicated in modulating host immune responses, contributing to viral pathogenesis [39,40] and its assembly at the ER–Golgi intermediate compartment [38]. Specific functions of the E gene and protein can vary within the *Coronaviridae* family in different virus types [41]; however, in-depth analyses of the diverse functions of either have not been performed for FCoV so far. This lack of knowledge is surprising considering the roles of the E protein in viral assembly, stability, and interaction with the host cell [35,36,37,38,39,40].

Given the central roles of the E and RdRP gene products, alongside the regulatory functions of the leader sequence, investigating their expression and potential differences between cats with FIP and those systemically infected with FCoV but without the disease could provide valuable insights into viral replication dynamics and their role in the disease. To explore this, we designed three real-time RT-qPCR assays: two targeting subgenomic mRNAs, to obtain information on the transcription of RdRp and E, and the third to detect both RdRP gRNA and mRNA, for quantifying virion production.

## 2. Materials and Methods

### 2.1. Study Population and Sampling

This study was performed on tissues from 46 cats with FIP, of which a large proportion had been included in previous studies [12,13,21,22]. All animals had been euthanized due to severe clinical disease and were subjected to a diagnostic postmortem examination. FIP was confirmed by gross and histological examination, in combination with immunohistology for FCoV antigen that detected the virus in macrophages of the granulomatous lesions, as previously described [20], as well as a routine diagnostic 7b FCoV RT-qPCR [42,43]. Samples from MLNs or from organs with confirmed FIP lesions were collected, immersed in RNAlater^®^ (Qiagen, Hilden, Germany), and stored at −80 °C until use. In addition, the MLN (collected and stored as per the tissues from cats with FIP and confirmed to carry the virus by the FCoV 7b RT-qPCR) from 10 cats without FIP were included; these had already been included in previous studies [12,13,21,22]. All cats, with and without FIP, had been subjected to the postmortem examination upon request and/or with full permission from the owners within 2 h after death. Detailed information on individual cases is provided in Supplementary Table S1.

### 2.2. RNA Extraction

One tissue sample per cat was examined, MLN or from an organ with lesions (liver, kidney, spleen). RNA was purified using the RNeasy Plus Minikit^®^ (Qiagen, Hilden, Germany) in duplicate. Briefly, samples were thawed on ice and approximately 10 mg of tissue was cut and transferred into a 2 mL tube containing a 1.4 mm diameter ceramic bead (Omni International, Kennesaw, GA, USA) and 600 µL of extraction buffer (Buffer RLT Plus) with β-mercaptoethanol (Sigma-Aldrich, St. Louis, MO, USA). Tissues were mechanically disrupted and homogenized using the Precellys 24 Tissue Homogenizer (Bertin Technologies SAS, Montigny-le-Bretonneux, France). Further steps were performed according to the manufacturer’s instructions. RNA was eluted in 30 µL of RNase-free water. The eluted RNA of each extraction was pooled for analysis. The RNA concentration was measured with the Qubit 4 Fluorometer (Thermo Fisher Scientific, Waltham, MA, USA) using the Qubit™ RNA BR Assay Kits (Thermo Fisher Scientific, Waltham, MA, USA) and in one sample (case 48, FCoV-infected cat without FIP) the Qubit™ RNA HS Assay Kit (Thermo Fisher Scientific) following the Qubit Assay Protocol.

### 2.3. FCoV 7b Quantitative Reverse Transcription Polymerase Chain Reaction (RT-qPCR) System

In cases where routine diagnostic 7b RT-qPCR had not yet been performed (cases 41–56), it was conducted as previously described [42], with described methodological adaptions [43].

### 2.4. RT-qPCR for FCoV E mRNA and RdRp mRNA and/or gRNA

Primer and probe design. Using the bioinformatics software Geneious Prime^®^ (version 2020.2.5), an integrated NCBI BLAST search for published whole genome sequences of FCoV was conducted. These sequences were imported and used to create a consensus sequence. Based on the consensus sequence, primers and probes of the regions of interest were designed accordingly. To avoid amplifying genomic RNA of the virus, the forward primers in the RdRp mRNA and E mRNA assay were designed to bind the viral leader sequence, the reverse primer binding to the beginning of the gene, as each subgenomic mRNA encompasses a common 5′ leader sequence that mirrors the genomic 5′ end, which serves as a conserved starting point for plus-strand RNA synthesis [26]. The primers and probe for the FCoV RdRp gRNA and mRNA combined assay were selected spanning the RdRp 5′ untranslated region and the beginning of the ORF. The final selected primers were analyzed using NCBI blast (https://blast.ncbi.nlm.nih.gov/Blast.cgi, accessed on 8 January 2021) to avoid primer binding for non-target feline genes. All designed primers and probes (Table 1) were then synthesized by Microsynth (Balgach, Switzerland).

Preparation of the RT-qPCR standards for the polymerase and envelope system. For each assay, sequence-specific RNA was synthesized and used to determine the viral RNA copy numbers. For the RdRp (“FCoV-P-Std”) and the E (“FCoV-E-Std”) mRNA assay, a synthetic gene was assembled from synthetic oligonucleotides and/or PCR products by a commercial provider (GeneArt Gene Synthesis services, Thermo Fisher Scientific). The corresponding plasmid maps are shown in Supplementary Figure S1.

Briefly, the fragment was inserted into a pcDNA3.1(+) vector backbone. The plasmid DNA was purified from transformed bacteria (*E. coli* K12 DH10B^Tm^T1R) and the concentration determined by UV spectroscopy. The final construct was verified by sequencing. The sequence identity within the insertion sites was 100%. The plasmids were linearized using the ApaI enzyme (Thermo Fisher Scientific) with incubation for 1 h at 37 °C and inactivation for 10 min at 80 °C. To check for successful complete plasmid linearization and to purify the linearized vector, 20 µL of each linearized plasmid DNA was electrophoretically analyzed on a 1.5% agarose gel. Distinct bands matching the molecular weight of the expected product (5935 bp) were visualized using the Fusion Solo S imaging system (Vilber Lourmat, Collégien, France), isolated from the gel, and the obtained DNA product was purified with the QIAquick^®^ Gel Extraction Kit (Qiagen) according to the provided purification protocol. The purified DNA was eluted twice with 30 µL RNase-free water.

RNA was synthesized from the linearized plasmids by in vitro transcription using the RiboMAX™ Large Scale RNA Production System T7 (Promega, Madison, WI, USA) from the T7 promotor according to the manufacturer’s protocol. The synthesized RNA was then treated with DNase (Promega) by incubating at 37 °C for 15 min to remove the DNA template.

RNA was further purified using the QIAamp RNA Blood Mini Kit (Qiagen) according to the manufacturer’s instructions. The RNA yield was determined using a Qubit 4 Fluorometer with the Qubit™ RNA BR Assay Kits. The RNA copy number for each standard was calculated based on the length of the synthetized RNA. RNA standards were prepared by a 10-fold serial dilution of the RNA templates in RNAse-free water containing 30 μg/mL carrier rRNA (Sigma-Aldrich).

RT-qPCR validation. Different forward and reverse primer pair concentrations were tested along with the corresponding RNA standards to find the optimal concentration resulting in the best efficiency of the RT-qPCR assays. An ABI Prism 7500Fast real-time PCR system and software v2.3 (Thermo Fisher Scientific) served for thermocycling and data analyses. After optimization, the two RdRp systems were combined in a duplex assay, with concentrations of 600 nM (forward primers) and 900 nM (reverse primers) each, while the E assay was conducted with both primers at a concentration of 900 nM. In both reaction systems, the probe concentration was 250 nM. Samples were tested in duplicate, using the AgPath-ID™ One-Step RT-PCR Reagents system (Thermo Fisher Scientific) and 5 μL of the RNA in a total reaction volume of 25 μL per well. The temperature profile comprised a reverse transcription step for 10 min at 45 °C, followed by initial denaturation and polymerase activation at 95 °C for 10 min, and finally 45 cycles of 15 s at 95 °C followed by 45 s at 60 °C. A standard curve from 10^0^–10^8^ copy numbers (in duplicate) and two negative controls were run on every plate to determine the viral RNA copy number.

### 2.5. Statistical Analysis

Statistical analyses were conducted using IBM SPSS Statistics, Version 29.0.2.0 (20). Prior to statistical comparisons, Shapiro–Wilk and Kolmogorov–Smirnov tests were performed to assess data distribution. Given that data were not normally distributed, non-parametric tests were employed throughout the analysis. To compare the viral loads in the different assays between groups (FIP and FCoV-infected without FIP), the Mann–Whitney U test was applied. Correlations between the results of the three RT-qPCR assays were evaluated using Spearman’s rho correlation analysis. To assess the consistency of viral load ratios among the three assays across cats in the FIP group, an intraclass correlation coefficient (ICC) analysis was performed. A two-way mixed-effects model was selected to evaluate consistency among the assays. This analysis included 37 cats that were valid for analysis. Detailed ICC values, confidence intervals, and exact viral load ratio values are presented in Supplementary Table S2.

Additionally, statistical analyses were conducted using RStudio 2023.09.0 + 463 (codenamed “Juniper”). Data were analyzed using the Friedman test to determine whether there were copy number differences among the three assays within each group. Following the Friedman test, pairwise comparisons were conducted using Dunn’s test to identify which specific assays differed from each other. The Dunn test was performed with a Bonferroni adjustment for multiple comparisons.

The Friedman test was conducted in R using the friedman.test() function. Subsequently, pairwise comparisons were performed with the dunn.test() function from the “dunn.test” package, setting the kw parameter to FALSE and applying the Bonferroni correction for the *p*-values.

Statistical significance was set at *p* < 0.05, and the results were reported, including Z-values and adjusted *p*-values for each pairwise comparison.

## 3. Results

### 3.1. Efficiency and Standard Curve Performance of the RdRp gRNA and mRNA, RdRp mRNA, and E mRNA Assays

The efficiency and R^2^ values (a measure of linearity in standard curves) for the three assays are summarized in Supplementary Table S3. All assays showed good linearity, with R^2^ values above 0.98. The RdRp gRNA and mRNA assay had lower efficiency (75.79%) than the RdRp mRNA and E mRNA assays (105.04% and 99.83%, respectively). These efficiency values for the RdRp gRNA and mRNA and RdRp mRNA assays were obtained from the duplex assays.

### 3.2. Variable RdRp gRNA and mRNA, RdRp mRNA, and E mRNA Levels in Samples of Cats with FIP and FCoV-Infected Cats Without FIP

The Ct values for the 7b RT-qPCR assay, which was applied to confirm FCoV infection, are summarized in Table S4. In cats with FIP, the Ct values obtained from MLN or FIP lesions in tissues had a broad range, from 13.77 to 38.39, with a median Ct of 21.76. In contrast, FCoV-infected cats without FIP, where MLNs were tested, showed comparably higher Ct values (range 29.21 to 39.84), with a median Ct of 37.45.

In cats with FIP, all three assays (RdRp gRNA and mRNA, RdRp mRNA, E mRNA) yielded positive results, though not consistently in all samples. In one cat (cat 17), the MLN sample was negative for all assays. The 7b RT-qPCR for this sample had resulted in a high Ct (38.39; Supplementary Table S4) corresponding to a very low viral load, and immunohistology for viral antigen performed on an adjacent tissue sample processed accordingly had yielded a negative result [20]. In addition to this sample, an additional six samples tested negative in the RdRp gRNA and mRNA assay, of which one was also negative in the RdRp mRNA assay, and four also negative in the E mRNA assay. Three further samples were only negative in the E mRNA assay. In total, 15.2% (7/46) of samples were negative in the RdRp gRNA and mRNA assay, 4.3% (2/46) in the RdRp mRNA, and 17.4% (8/46) in the E mRNA assay, respectively. The copy numbers of viral RNA varied across all assays. Overall, the RdRp gRNA and mRNA assay copy numbers ranged from non-detectable (0 copies/ng of total RNA) up to 4821 copies/ng of total RNA, with a median of 106.66. The RdRp mRNA assay had a similarly wide range, from 0 to 7555 copies/ng of total RNA (mean = 125.82), while the E mRNA assay yielded lower copy numbers, ranging from 0 to 515 copies/ng of total RNA (mean: 0.47). The results obtained for individual animals are shown in Supplementary Table S4, while the Ct values of all assays (RdRp gRNA and mRNA, RdRp mRNA, E mRNA, and 7b RT-qPCR) are graphically summarized in Supplementary Figure S2. The differences in copy numbers for the three assays were significant (*p* < 0.001) within the FIP group. The pairwise comparisons using the Dunn posttest with Bonferroni adjustment revealed significant differences between the E mRNA assay and both the RdRp gRNA and mRNA and RdRp mRNA assays. These last assays did not differ significantly from each other (Supplementary Table S5A,B). In the ICC analysis of the 37 cats valid for this analysis, a moderate degree of consistency in viral load ratios among the three assays was observed in samples from cats with FIP. The exact values for the viral load ratios, along with detailed ICC values and confidence intervals, are provided in Supplementary Table S6.

In the FCoV-infected cats without FIP, in line with the higher 7b Ct values, overall, more samples tested negative than positive in all three assays. In six samples (60%), all three assays were negative, in another two, only RdRp mRNA was detected, of the remaining two, one tested positive in all three assays while the other was positive in the RdRP mRNA and the E mRNA assay (Supplementary Table S4). When detected, the RdRp gRNA and mRNA copy numbers reached a maximum of 12.9 copies/ng of total RNA (mean = 0); the RdRp mRNA assay copy numbers had a maximum of 45.1 copies/ng of total RNA (mean = 0); and the E mRNA assay results did not go above 0.01 copies/ng of total RNA (mean = 0). The results for each animal are shown in Supplementary Table S4. To evaluate differences in copy numbers among the assays in the FCoV-infected cats, a Friedman test was conducted, followed by Dunn’s test with Bonferroni adjustment. The analysis did not reveal any significant differences between the assays (Supplementary Table S2A,B). Given the high number of negative assay results in this group, comparisons of the assay copy number ratios were not carried out.

### 3.3. FIP Lesions Are Associated with Significantly Higher RdRp and E RNA Loads, in Line with Higher Viral Loads

**Figure 1:**
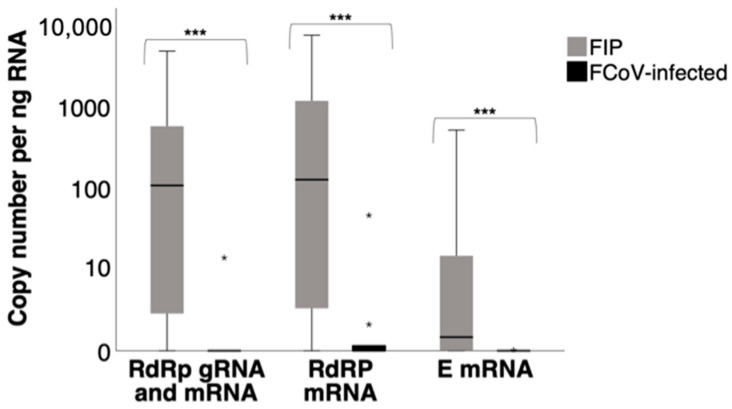
Comparison of viral RNA levels between cats with FIP (“FIP”; tissue samples with FIP lesions) and FCoV-positive cats without FIP (“FCoV-infected”; MLN samples) using three RT-qPCR assays. The box plots represent the distribution of RNA copy numbers for each group across three different assays: RdRp gRNA and mRNA, RdRp mRNA, and E mRNA. The central line in each box indicates the median, while the box itself represents the interquartile range (IQR: 25th to 75th percentiles). Whiskers extend to the smallest and largest values within 1.5 times the IQR, and any data points outside this range are shown as outliers (*). Copy numbers (log scale) per ng RNA. Mann–Whitney U test. ***: asymptotic significance <0.001.

In line with the higher viral loads determined in the 7b RT-qPCR assay in cats with FIP compared to FCoV-infected cats, all three assays (RdRp gRNA and mRNA, RdRp mRNA, E mRNA) yielded significantly higher copy numbers (*p* < 0.001) in the samples from the MLN and/or tissue lesions of cats with FIP than in the MLN of FCoV-infected cats without FIP (Figure 1). The detailed results regarding the copy numbers are provided in Supplementary Table S4; the results of the Mann–Whitney U test are provided in Supplementary Table S7.

### 3.4. Positive Correlation Between RdRp and E RT-qPCR Assays with FCoV Infection Regardless of Disease

**Figure 2:**
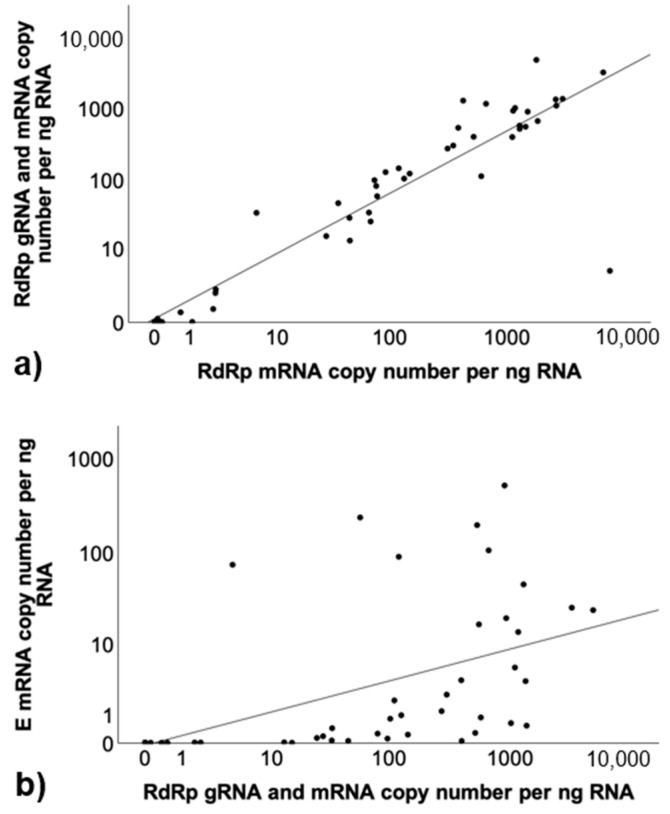

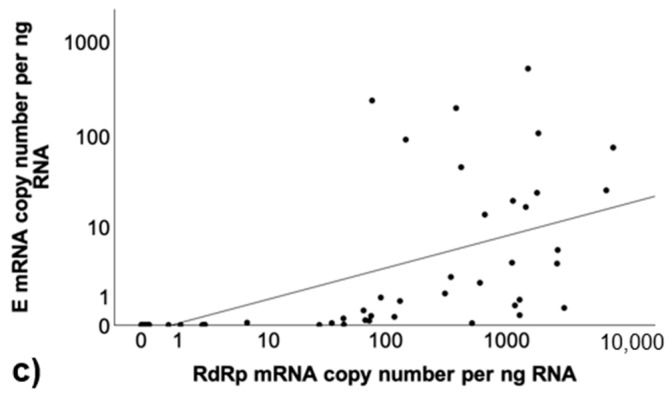
Scatter plot diagrams for copy number correlations. RdRp gRNA and mRNA with RdRp mRNA copy number correlation in (**a**), RdRp gRNA and mRNA with E mRNA in (**b**), and RdRp mRNA with E mRNA in (**c**). These plots display the comparison of copy numbers between the assays across all samples, regardless of group. Individual data points are shown, with a guiding line representing the line of best fit, illustrating the correlation between the results of the two assays. Copy numbers (log scale) per ng RNA.

A correlation analysis was conducted to examine the relationships between the three assays regardless of disease. This analysis revealed strong positive correlations among the RdRp gRNA and mRNA, RdRp mRNA, and E mRNA assays, indicating that when RNA copy numbers are higher in one assay, they also tend to be higher in the others. These results are graphically illustrated in Figure 2; the detailed results of the correlation analysis are shown in Supplementary Table S8.

Due to the high rate of negative assay results in the FCoV-infected cats without FIP, the correlation analysis was conducted separately for both groups. In the FIP cats, the copy numbers of the three assays correlated strongly (Supplementary Table S7); the correlations were apparent but overall weaker in the FCoV-infected cats without FIP. In the latter, while the E mRNA assay copy numbers significantly correlated with RdRp gRNA and mRNA and RdRp mRNA, the RdRp mRNA and RdRp gRNA and mRNA copy numbers did not significantly correlate with each other (correlation factor: 0.588) (Supplementary Table S8).

## 4. Discussion

It is accepted that, even when the clinical picture does not indicate systemic disease, infection of cats with FCoV generally leads to viremia and spread of the virus into tissues [44,45,46]. In most cats, only low-level viremia is detectable and progression to FIP may not occur [45]; in cats with fulminant FIP, high viral RNA levels are generally detected [47]. In previous studies, we have detected FCoV at low levels in the MLN of cats without FIP, choosing this tissue as it represents a likely portal of entry for an enteric virus that is taken up and distributed by macrophages and monocytes, and characterized the effect of the virus on the tissue [21,22]. The present study made use of these infected but unaltered MLNs and of MLNs and tissues with FIP lesions, to investigate whether viral genes are differentially expressed in infected tissues with and without the typical granulomatous lesions that carry virus-laden macrophages. It is the first to attempt the quantification and correlation of the gene expression of relevant FCoV proteins, the viral polymerase (RdRp) and the envelope (E) structural protein, in FCoV infection and FIP. Three quantitative RT-qPCR assays were developed, two targeting the leader sequence, allowing the quantification of RdRp and E mRNA levels, and for the third, RdRp gRNA and mRNA combined. These were applied to the MLN of FCoV-infected cats without FIP (tested positive for viral RNA, in a well-established diagnostic PCR targeting the non-structural 7b gene at the 3′ end of the FCoV genome), and the MLN and tissue lesions of cats with FIP, with the aim to evaluate the quantitative transcriptional differences between the groups and potential correlations between RdRp and structural gene mRNA expression.

The three RT-qPCR assays developed in this study consistently demonstrated significant differences in viral RNA levels between FIP and non-FIP cats, with lower levels in the latter. The variable viral RNA levels across all three assays (RdRp gRNA and mRNA, RdRp mRNA, and E mRNA) in the samples from the cats with FIP demonstrated substantial heterogeneity in viral differential expression in FIP lesions. In line with these results, the Ct values of the 7b RT-qPCR assay also varied substantially between the samples from cats with FIP, but were sometimes very low (<14), consistent with high viral loads. In the MLN of FCoV-infected cats without FIP, much lower viral loads were detected. These results suggest a correlation of the 7b RT-qPCR assay and the newly developed assays. Overall, the findings are in agreement with the results of an older study, which demonstrated that cats with FIP exhibit significantly higher viral loads than healthy infected cats in tissue samples of the spleen, mesenteric lymph nodes, and bone marrow [13,47]. Differences in virus load in lesions and in affected cats in general may be attributed to several factors, among these, a variable immune response. For example, lower IL-12 levels can impede an effective cellular immune response, which could lead to the enhanced monocyte/macrophage activation typically associated with FIP [48]. This is particularly relevant, as macrophages are the target cells in FIP, with the virus replicating efficiently within them, sustaining an increased mutation rate and thus allowing rapid dissemination of the virulent virus throughout the body [49,50].

Disease and lesion duration prior to euthanasia may also have affected the results as the dominating cell populations in FIP lesions change over time. Initiated and developed based on the emigration of monocytes and accumulation of (infected) macrophages [50], lesions then gather progressively more B cells that later differentiate into plasma cells; with replacement of the macrophages, less viral antigen is expressed [20,46].

Only one sample from an FIP cat tested negative in all three assays (cat 17). The 7b RT-qPCR had yielded a very high Ct value of 38.39. This suggests that the viral load in this particular sample was very low, likely explaining the failure of the other assays. Indeed, in this particular lymph node, we also failed to detect FIP lesions and viral antigen.

We found significantly lower copy numbers for E mRNA than for both RdRp assays. This difference could be attributed to the requirement for only small amounts of E protein during construction of the virion [35].

In contrast, viral RNAs were mainly undetectable or present only at very low levels in the MLN of the FCoV-infected cats without FIP. This aligns with the high 7b RT-qPCR Ct values. Our findings suggest consistent virion production in association with FIP lesions with only very limited virion production with systemic FCoV infection alone. This likely reflects both the increased macrophage density in lesions (as the infected cell type) and the higher replication potential within these cells of the pathogenic biotype.

The low viral mRNA concentrations in non-FIP cats are in line with the expectation that systemic FCoV infection without disease is associated with no or only minimal active viral replication. This is consistent with the results of an earlier study, where FCoV mRNA could be detected in the blood of 46% of cats with FIP vs. 5% of healthy cats without FIP in the same household or cattery as the cats suffering from FIP [51].

Only one FCoV-infected cat without FIP exhibited transcription levels in the MLN comparable to those in FIP lesions. This sample also showed a notably lower 7b RT-qPCR Ct value (29.2) than the median Ct value of 37.45 across the rest of the control group. This cat had interstitial pneumonia of unknown cause; it is possible that it was also in a very early phase of FCoV infection or on the verge of developing FIP.

The three assays showed strong and significant correlations in FIP lesions. The high correlation coefficients between the RdRp mRNA and E mRNA assays indicate that the different target regions are transcribed in a ratio that is comparable across lesions and cats. This could be confirmed by an intraclass correlation coefficient analysis. In the FCoV-infected cats without FIP, the correlations were weaker, which could be attributed to the high frequency of negative results. The low number of cases with detectable viral RNA in this group reduced the statistical power of the correlation analysis. Despite this, there were still significant correlations between the RdRp gRNA and mRNA and E mRNA assay as well as between the RdRp mRNA and E mRNA assay. The ICC analysis in this group and the comparison of this analysis with the FIP group could not be conducted due to the high number of negative values. For this reason, it was not possible to determine a potential differential expression of the viral genes between the two groups.

The copy number of the RdRp gRNA and mRNA assay was generally lower than that of the RdRp mRNA assay, which is unexpected since the RdRp gRNA and mRNA assay should include both genomic RNA and messenger RNA, while the RdRp leader assay targets only mRNA [24]. A possible explanation for this discrepancy could lie in differences in efficiency between the assays. The lower efficiency of the RdRp gRNA and mRNA assay (75.79%) may contribute to reduced amplification compared to the RdRp mRNA assay with its higher efficiency (105.04%) and likely greater sensitivity under the given conditions.

While the results of the present study are encouraging, several limitations need to be addressed. The sample size, particularly for the FCoV-infected cats without FIP, was relatively small, which may impact the generalizability of the findings. Additionally, the high rate of negative results in the FCoV-infected group limited the correlation analysis. Possible future studies should include larger cohorts to validate these findings and explore the use of these assays in different clinical settings.

Moreover, it would be beneficial to investigate the potential of these assays in monitoring treatment response and disease progression in FIP cats. Longitudinal studies could provide insights into differential expression over time and in response to therapeutic interventions.

## Figures and Tables

**Table 1:** Primers and probes for the qPCR for RNA-dependent RNA polymerase (RdRp) gRNA and mRNA, RdRp mRNA, and envelope (E) mRNA.

Target	Primers, Probes	Nucleotide Sequences *	Amplicon Length
RdRp gRNA and mRNA	Forward primer	5′-AAG TCG TCT AGT ATT AGC TGC GG-3′	107 bp
	Reverse primer	5′-TTG CTT GGA ACT CAT TCT CCT GG-3′	
	Probe	5′-YY-TAG ACC GGG TTT CGT CCT GTG ATC TCC CTC-BHQ-1-3′	
RdRp Leader (RdRp mRNA)	Forward primer	5′-GGA CAC CAA CTC GAA CTA AAC GA-3′	165 bp
	Reverse primer	5′-CCG CAG CTA ATA CTA GAC GAC TT-3′	
	Probe	5′-6FAM-TTT GGC AAT CAC TCC TTG GAA CGG GGT TGA-BHQ-1-3′	
E Leader (E mRNA)	Forward primer	5′-TAC GGA CAC CAA CTC GAA CTA AA-3′	122 bp
	Reverse primer	5′-AGA AGA AGC CGC TAA CAA CCA TA-3′	
	Probe	5′-YY-AAG AGC GTG TCT ATG ATG TTT CCT AGG GCA-BHQ-1-3′	

*: YY: Yakima Yellow; BHQ-1: Black Hole Quencher 1; 6FAM: 6-Carboxyfluorescein.

## Data Availability

Data are contained within the article and Supplementary Materials.

## Supplementary Materials

The following supporting information can be downloaded at: https://www.mdpi.com/article/10.3390/v17020279/s1, Table S1: Animals included into the study, with information on the type of sample (organ material) examined. Table S2: Friedman Test results FCoV-infected cats without FIP, with the test statistics (A) and the Dunn test with Bonferroni adjustment (B). Table S3: Efficiency and standard curve performance of the RdRp gRNA and mRNA, RdRp mRNA and E mRNA assays. Table S4: RT-qPCR copy numbers, provided as copy numbers per nanogram of RNA normalized to the RNA concentration of each sample and corresponding Ct values. For the 7b RT-qPCR, only the Ct values are provided. Table S5: Friedman Test results obtained from cats with FIP, with the test statistics (A) and the Dunn test with Bonferroni adjustment (B). Table S6: Copy number ratios of the RdRp gRNA and mRNA, RdRp mRNA, E mRNA assay (A) and Intraclass Correlation Coefficient (ICC) of FIP assay Ratios (B). Table S7: Mann-Whitney U test group comparison of cats with FIP and FCoV-infected cats without FIP. Table S8: Spearman’s rho correlation analysis. Figure S1: Plasmid maps of the synthetic plasmids with synthetic envelope (above) and polymerase gene (below) from GeneArt Gene Synthesis services, Thermo Fisher Scientific. Figure S2: Plot of the Ct values of RdRp gRNA and mRNA, RdRp mRNA, E mRNA, and 7bq PCR in individual cats.

## References

[1] International Committee on Taxonomy of Viruses (ICTV) Family: Coronaviridae. Chapter Version: ICTV Ninth Report; 2009 Taxonomy Release. https://ictv.global/taxonomy/.

[2] Meli M., Kipar A., Müller C., Jenal K., Gönczi E., Glaus T., Lutz H. (2004). High Viral Loads Despite Absence of Clinical and Pathological Findings in Cats Experimentally Infected with Feline Coronavirus (FCoV) Type I and in Naturally FCoV-Infected Cats. J. Feline Med. Surg..

[3] Drechsler Y., Alcaraz A., Bossong F.J., Collisson E.W., Diniz P.P. (2011). Feline Coronavirus in Multicat Environments. Vet. Clin. N. Am. Small Anim. Pract..

[4] Felten S., Klein-Richers U., Hofmann-Lehmann R., Bergmann M., Unterer S., Leutenegger C.M., Hartmann K. (2020). Correlation of feline coronavirus shedding in feces with coronavirus antibody titer. Pathogens.

[5] Klein-Richers U., Hartmann K., Hofmann-Lehmann R., Unterer S., Bergmann M., Rieger A., Leutenegger C., Pantchev N., Balzer J., Felten S. (2020). Prevalence of feline coronavirus shedding in German catteries and associated risk factors. Viruses.

[6] Pedersen N.C. (1976). Serologic studies of naturally occurring feline infectious peritonitis. Am. J. Vet. Res..

[7] Addie D., Belak S., Boucraut-Baralon C., Egberink H., Frymus T., Gruffydd-Jones T., Hartmann K., Hosie M.J., Lloret A., Lutz H. (2009). Feline infectious peritonitis. ABCD guidelines on prevention and management. J. Feline Med. Surg..

[8] Addie D.D., Toth S., Murray G.D., Jarrett O. (1995). Risk of feline infectious peritonitis in cats naturally infected with feline coronavirus. Am. J. Vet. Res..

[9] Chang H.W., Egberink H.F., Halpin R., Spiro D.J., Rottier P.J.M. (2012). Spike protein fusion peptide and feline coronavirus virulence. Emerg. Infect. Dis..

[10] Licitra B.N., Millet J.K., Regan A.D., Whittaker G.R. (2013). Mutation in spike protein cleavage site and pathogenesis of feline coronavirus. Emerg. Infect. Dis..

[11] Jaimes J.A., Whittaker G.R. (2017). Feline coronavirus: Insights into viral pathogenesis based on the spike protein structure and function. Virology.

[12] Porter E., Tasker S., Day M.J., Harley R., Kipar A., Siddell S.G., Helps C.R. (2014). Amino acid changes in the spike protein of feline coronavirus correlate with systemic spread of virus from the intestine and not with feline infectious peritonitis. Vet. Res..

[13] Barker E.N., Stranieri A., Helps C.R., Porter E., Davidson A.D., Duchatel J.P., Kipar A. (2017). Limitations of Using Feline Coronavirus Spike Protein Gene Mutations to Diagnose Feline Infectious Peritonitis. Vet. Res..

[14] Jähne S., Felten S., Bergmann M., Erber K., Matiasek K., Meli M.L., Hofmann-Lehmann R., Hartmann K. (2022). Detection of Feline Coronavirus Variants in Cats without Feline Infectious Peritonitis. Viruses.

[15] Emmler L., Felten S., Matiasek K., Balzer H.J., Pantchev N., Leutenegger C., Hartmann K. (2020). Feline Coronavirus with and without Spike Gene Mutations Detected by Real-Time RT-PCRs in Cats with Feline Infectious Peritonitis. J. Feline Med. Surg..

[16] Golovko L., Lyons L.A., Liu H., Sørensen A., Wehnert S., Pedersen N.C. (2013). Genetic Susceptibility to Feline Infectious Peritonitis in Birman Cats. Virus Res..

[17] Pesteanu-Somogyi L.D., Radzai C., Pressler B.M. (2006). Prevalence of Feline Infectious Peritonitis in Specific Cat Breeds. J. Feline Med. Surg..

[18] Pedersen N.C. (1987). Virologic and Immunologic Aspects of Feline Infectious Peritonitis Virus Infection. Adv. Exp. Med. Biol..

[19] Weiss R.C., Scott F.W. (1981). Pathogenesis of Feline Infectious Peritonitis: Pathologic Changes and Immunofluorescence. Am. J. Vet. Res..

[20] Kipar A., Bellmann S., Kremendahl J., Köhler K., Reinacher M. (1998). Cellular Composition, Coronavirus Antigen Expression and Production of Specific Antibodies in Lesions in Feline Infectious Peritonitis. Vet. Immunol. Immunopathol..

[21] Malbon A.J., Meli M.L., Barker E.N., Davidson A.D., Tasker S., Kipar A. (2019). Inflammatory Mediators in the Mesenteric Lymph Nodes, Site of a Possible Intermediate Phase in the Immune Response to Feline Coronavirus and the Pathogenesis of Feline Infectious Peritonitis. J. Comp. Pathol..

[22] Malbon A.J., Russo G., Burgener C.J., Barker E.N., Meli M.L., Tasker S., Kipar A. (2020). The Effect of Natural Feline Coronavirus Infection on the Host Immune Response: A Whole-Transcriptome Analysis of the Mesenteric Lymph Nodes in Cats with and without Feline Infectious Peritonitis. Pathogens.

[23] Fehr A.R., Perlman S., Maier H., Bickerton E., Britton P. (2015). Coronaviruses: An Overview of Their Replication and Pathogenesis. Coronaviruses.

[24] V’kovski P., Kratzel A., Steiner S., Stalder H., Thiel V. (2021). Coronavirus biology and replication: Implications for SARS-CoV-2. Nat. Rev. Mol. Microbiol..

[25] Sawicki S.G., Sawicki D.L., Siddell S.G. (2007). A Contemporary View of Coronavirus Transcription. J. Virol..

[26] Malone B., Urakova N., Snijder E.J., Campbell E.A. (2022). Structures and Functions of Coronavirus Replication–Transcription Complexes and Their Relevance for SARS-CoV-2 Drug Design. Nat. Rev. Mol. Cell Biol..

[27] Yin X., Popa H., Stapon A., Bouda E., Garcia-Diaz M. (2023). Fidelity of Ribonucleotide Incorporation by the SARS-CoV-2 Replication Complex. J. Mol. Biol..

[28] Stevens L.J., Pruijssers A.J., Lee H.W., Gordon C.J., Tchesnokov E.P., Gribble J., George A.S., Hughes T.M., Lu X., Li J. (2022). Mutations in the SARS-CoV-2 RNA-Dependent RNA Polymerase Confer Resistance to Remdesivir by Distinct Mechanisms. Sci. Transl. Med..

[29] Gao Y.Y., Liang X.Y., Wang Q., Zhang S., Zhao H., Wang K., Hu G.X., Liu W.J., Gao F.S. (2022). Mind the Feline Coronavirus: Comparison with SARS-CoV-2. Gene.

[30] Bos E.C., Luytjes W., van der Meulen H.V., Koerten H.K., Spaan W.J. (1996). The Production of Recombinant Infectious DI-Particles of a Murine Coronavirus in the Absence of Helper Virus. Virology.

[31] Lu W., Zhao Z., Huang Y.-W., Wang B. (2022). A Systematic Review of Virus-Like Particles of Coronavirus: Assembly, Generation, Chimerism and Their Application in Basic Research and in the Clinic. Int. J. Biol. Macromol..

[32] Vennema H., Godeke G.J., Rossen J.W., Voorhout W.F., Horzinek M.C., Opstelten D.J., Rottier P.J. (1996). Nucleocapsid-Independent Assembly of Coronavirus-Like Particles by Co-Expression of Viral Envelope Protein Genes. Eur. Mol. Biol. Organ. J..

[33] Takano T., Nakano K., Doki T., Hohdatsu T. (2015). Differential Effects of Viroporin Inhibitors against Feline Infectious Peritonitis Virus Serotypes I and II. Arch. Virol..

[34] Venkatagopalan P., Daskalova S.M., Lopez L.A., Dolezal K.A., Hogue B.G. (2015). Coronavirus Envelope (E) Protein Remains at the Site of Assembly. Virology.

[35] Fischer F., Stegen C.F., Masters P.S., Samsonoff W.A. (1998). Analysis of Constructed E Gene Mutants of Mouse Hepatitis Virus Confirms a Pivotal Role for E Protein in Coronavirus Assembly. J. Virol..

[36] Lim K., Liu D. (2001). The Missing Link in Coronavirus Assembly: Retention of the Avian Coronavirus Infectious Bronchitis Virus Envelope Protein in the Pre-Golgi Compartments and Physical Interaction between the Envelope and Membrane Proteins. J. Biol. Chem..

[37] Corse E., Machamer C.E. (2000). Infectious Bronchitis Virus Envelope Protein Is Targeted to the Golgi Complex and Directs Release of Virus-Like Particles. J. Virol..

[38] Corse E., Machamer C.E. (2003). The Cytoplasmic Tails of Infectious Bronchitis Virus Envelope and Membrane Proteins Mediate Their Interaction. Virology.

[39] Ruch T.R., Machamer C.E. (2012). The Coronavirus Envelope Protein: Assembly and Beyond. Viruses.

[40] Zhou S., Lv P., Li M., Chen Z., Xin H., Reilly S., Zhang X. (2023). SARS-CoV-2 Envelope Protein: Pathogenesis and Potential Therapeutic Development. Biomed. Pharmacother..

[41] DeDiego M.L., Álvarez E., Almazán F., Rejas M.T., Lamirande E., Roberts A., Shieh W., Zaki S.R., Subbarao K., Enjuanes L. (2007). A Severe Acute Respiratory Syndrome Coronavirus That Lacks the E Gene Is Attenuated In Vitro and In Vivo. J. Virol..

[42] Gut M., Leutenegger C.M., Huder J.B., Pedersen N.C., Lutz H. (1999). One-Tube Fluorogenic Reverse Transcription-Polymerase Chain Reaction for the Quantitation of Feline Coronaviruses. J. Virol. Methods.

[43] Zuzzi-Krebitz A.M., Buchta K., Bergmann M., Krentz D., Zwicklbauer K., Dorsch R., Wess G., Fischer A., Matiasek K., Hönl A. (2024). Short Treatment of 42 Days with Oral GS-441524 Results in Equal Efficacy as the Recommended 84-Day Treatment in Cats Suffering from Feline Infectious Peritonitis with Effusion—A Prospective Randomized Controlled Study. Viruses.

[44] Lutz M., Meli M.L., Wess G., Hienz S., Riond B., Hartmann K., Kipar A. (2020). Feline Coronavirus Viral Sequences of Systemically Infected Healthy Cats Lack Gene Mutations Previously Linked to the Development of Feline Infectious Peritonitis. Pathogens.

[45] Gunn-Moore D.A., Gruffydd-Jones T.J., Harbour D.A. (1998). Detection of feline coronaviruses by culture and reverse transcriptase-polymerase chain reaction of blood samples from healthy cats and cats with clinical feline infectious peritonitis. Vet. Microbiol..

[46] Kipar A., Meli M.L. (2014). Feline Infectious Peritonitis: Still an Enigma?. Vet. Pathol..

[47] Kipar A., Baptiste K., Barth A., Reinacher M. (2006). Natural feline coronavirus infection: Cats with feline infectious peritonitis exhibit significantly higher viral loads than healthy infected cats. J. Feline Med. Surg..

[48] Kipar A., Meli M.L., Failing K., Euler T., Gomes-Keller M.A., Schwartz D., Lutz H., Reinacher M. (2006). Natural feline coronavirus infection: Differences in cytokine patterns in association with the outcome of infection. Vet. Immunol. Immunopathol..

[49] Malbon A.J., Michalopoulou E., Meli M.L., Barker E.N., Tasker S., Baptiste K., Kipar A. (2020). Colony Stimulating Factors in Early Feline Infectious Peritonitis Virus Infection of Monocytes and in End Stage Feline Infectious Peritonitis; A Combined In Vivo and In Vitro Approach. Pathogens.

[50] Kipar A., May H., Menger S., Weber M., Leukert W., Reinacher M. (2005). Morphologic Features and Development of Granulomatous Vasculitis in Feline Infectious Peritonitis. Vet. Pathol..

[51] Simons F.A., Vennema H., Rofina J.E., Pol J.M., Horzinek M.C., Rottier P.J., Egberink H.F. (2005). A mRNA PCR for the Diagnosis of Feline Infectious Peritonitis. J. Virol. Methods.

