# Supplementary material for: Deciphering Viral Replication Dynamics in Feline Infectious Peritonitis: A Quantitative Approach
Source: Viruses. 2025 Feb 18;17(2):279. doi: 10.3390/v17020279 (PMC11860597; doi:10.3390/v17020279)
Supplement: Supplementary file 1 [file viruses-17-00279-s001.zip › viruses-3454096-supplementary.pdf]

Supplementary material

Table S1. Animals included into the study, with information on the type of sample (organ material) examined.

A. Cats with FIP

Case No	Breed	Sex	Age	Organ material
1	NR	NR	3 y	MLN
2	Burmese	M	3 m	MLN
3	Abyssinian	F	4 m	MLN
4	DSH	NR	NR	MLN
5	DSH	NR	5 m	MLN
6	Siamese	NR	1 y	MLN
7	BSH	MN	10 m	MLN
8	DSH	MN	2 y	MLN
9	Siamese	MN	3 y	MLN
10	Birman	MN	12 y	MLN
11	BSH	FN	1 y	MLN
12	DSH	MN	2 y	MLN
13	Oriental	M	3 y	MLN
14	Birman	M	8 m	MLN
15	Ragdoll	FN	10 m	MLN
16	BSH	MN	2 y	MLN
17	DSH	F	6 m	MLN
18	DSH	NR	1 y	MLN
19	DSH	NR	4 m	MLN
20	DSH	NR	7 m	MLN
21	DSH	M	NR	MLN
22	DSH	FN	10 y	MLN
23	Norwegian Forest Cat	MN	8 m	MLN
24	DSH	M	4 m	MLN
25	DSH	MN	1.5 y	MLN
26	Maine Coon	MN	1 y	MLN
27	DSH	MN	6 m	MLN
28	DSH	F	4 m	MLN
29	BSH	MN	6 y	MLN
30	Persian	F	5 m	MLN
31	DSH	M	1 y	MLN
32	DSH	M	1 y	MLN
33	NR	NR	NR	MLN
34	NR	NR	NR	MLN
35	NR	NR	NR	Kidney
36	NR	NR	NR	Spleen
37	NR	NR	NR	Spleen
38	NR	NR	NR	Spleen
39	Sphynx	F	1 y 6 m	Kidney
40	DSH	M	2 y	Kidney
41	BSH	M	8 m	MLN

42	DSH	MN	1 y 7m	Liver
43	DSH	M	3 y	Kidney
44	NR	M	4 y	Kidney
45	Persian	M	14 y	Kidney
46	Persian	M	2 y	Kidney

Abbreviations: F – female; M – male; MN – male neutered; FM – female neutred; m – month(s); MLN – mesenteric lymph nodes; y – year(s); NR – not recorded; DSH - Domestic Shorthair; BSH – British Shorthair

B. FCoV-infected cats without FIP. From all animals, a sample from the mesenteric lymph node was examined, and FCoV infection was confirmed by RT-qPCR for the FCoV 7b gene [53].

Case No	Breed	Sex	Age	Diagnosis
47	Maine Coon	NR	1 y	Sclerosing encapsulating peritonitis
48	DSH	MN	3 y	Lethargy, weight loss, anaemia
49	DSH	MN	10 y	Diabetes mellitus
50	Ragdoll	M	4 m	Severe interstitial pneumonia
51	Havana	FN	4 y	Nasal lymphoma
52	DSH	FN	10 y	Round cell neoplasia
53	DSH	MN	8 y	Pleural effusion (PCR neg)
54	DSH	FN	18 y	Chronic renal disease
55	DSH	MN	10 y	Lymphoma
56	DSH	F	NR	Anaesthetic death

Abbreviations: F – female; M – male; MN – male neutered; FM – female neutred; m – month(s); y - year(s); NR – not recorded; DSH - Domestic Shorthair

Table S2. Friedman Test results FCoV-infected cats without FIP, with the test statistics (**A**) and the Dunn test with Bonferroni adjustment (**B**).

A. Friedman Test for the FCoV-infected cats without FIP to compare median ranks across multiple assays (RdRP gRNA and mRNA, RdRP mRNA, and E mRNA).

Assay	Mean Rank
RdRP gRNA and mRNA	1.80
RdRP mRNA	2.40
E mRNA	1.80

Test Statistics of Friedman Test	
N	10
Chi-Square	6.857
df	2
Asymptotic Significance	0.032

B. Friedman Test results with Dunn test and Bonferroni adjustment.

Comparison	Z value	P value adjusted
RdRp gRNA and mRNA vs. RdRp mRNA		0.1725
RdRp gRNA and mRNA vs. E mRNA	-0.2741	1.0000
RdRp mRNA vs. E mRNA	1.3021	0.2893

Table S3. Efficiency and standard curve performance of the RdRp gRNA and mRNA, RdRp mRNA and E mRNA assays.

Assay	R^2 value	Efficiency (%)
RdRP gRNA and mRNA	0.995	75.79
RdRP mRNA	0.981	105.04
E mRNA	0.999	99.83

Table S4. RT-qPCR copy numbers, provided as copy numbers per nanogram of RNA normalized to the RNA concentration of each sample and corresponding Ct values. For the 7b RT-qPCR, only the Ct values are provided.

Case No	RdRp gRNA and mRNA		RdRp mRNA		E mRNA		7b RT-qPCR
	Copy No	Ct	Copy No	Ct	Copy No	Ct	Ct
**Cats with FIP**							
1	0	UD	0	UD	0.005	38.092	37.344
2	516.353	23.68	1285.454	19.714	0.27	31.057	20.807
3	574.387	24.266	1284.264	20.378	0.848	31.075	17.575
4	102.45	29.444	132.498	25.498	0.792	31.638	29.221
5	928.643	23.178	1131.756	20.39	19.591	25.637	19.791
6	396.031	25.018	1112.902	20.679	3.561	28.183	23.604
7	4821.18	20.34	1786.531	19.821	24.037	25.831	15.12
8	15.088	30.248	27.976	25.703	0.001	40.848	22.413
9	0	UD	0.084	35.183	0	UD	34.829
10	1.857	35.787	2.321	31.013	0.006	38.325	22.035
11	3217.58	24.112	6630.427	20.365	25.572	27.664	17.206
12	553.827	23.886	1444.683	19.831	16.702	25.712	17.964
13	120.682	28.456	147.842	24.839	90.481	24.737	19.634
14	0	UD	0.045	36.438	0	UD	34.741
15	57.539	32.173	77.67	27.806	236.118	25.207	15.762
16	126.478	29.002	91.843	26.045	0.951	31.378	18.07
17	0	UD	0	UD	0	UD	38.393
18	0.11	39.068	0.06	35.306	0	UD	28.995
19	80.48	28.07	76.248	24.89	0.248	31.927	21.979
20	28.016	29.013	44.736	24.901	0.172	31.695	20.605
21	24.826	30.067	68.201	24.928	0.123	32.762	20.819
22	1340.76	22.852	2611.608	19.387	3.464	28.229	19.844
23	665.594	24.775	1832.123	20.508	105.829	24.08	20.685
24	33.447	29.037	65.968	24.589	0.424	30.742	25.482
25	1016.185	22.617	1175.98	19.99	0.61	30.113	20.497
26	1366.592	23.11	2988.88	19.349	0.515	31.59	20.659
27	300.404	24.906	348.931	22.599	2.217	29.21	25.142
28	399.253	23.636	521.234	21.289	0.045	34.11	23.092

29	1165.651	22.706	664.161	21.762	13.708	26.809	22.117
30	272.655	24.581	311.973	22.387	1.154	29.744	21.858
31	534.911	23.2	385.036	21.849	196.164	22.345	17.225
32	900.464	23.386	1505.415	20.592	515.074	21.824	16.895
33	1.552	33.991	2.298	30.528	0.006	37.299	24.608
34	143.442	25.563	119.146	23.733	0.218	31.942	20.113
35	1095.653	24.11	2641.684	20.688	5.212	29.191	16.429
36	0	UD	0.113	36.194	0.003	39.164	30.474
37	0	UD	0.038	38.292	0	UD	33.541
38	4.228	32.363	7553.689	17.483	74.375	24.133	13.767
39	0.364	39.045	0.666	34.468	0	UD	29.003
40	0.521	38.739	2.157	33.124	0	UD	30.46
41	0	UD	0.022	39.201	0	UD	31.653
42	45.708	30.075	35.676	27.918	0.047	36.116	23.515
43	110.87	30.715	605.314	25.452	1.795	32.694	21.109
44	33.197	29.293	6.38	29.472	0.057	34.728	23.636
45	1290.175	23.225	423.958	23.142	45.716	25.665	14.346
46	97.251	26.617	73.631	24.758	0.106	33.198	21.66
**FCoV-infected cats without FIP**							
47	0	UD	0	UD	0	UD	39.796
48	0	UD	0	UD	0	UD	37.81
49	0	UD	0	UD	0	UD	37.29
50	12.899	29.869	45.094	24.408	0.011	35.024	29.206
51	0	UD	0	UD	0	UD	37.602
52	0	UD	0	UD	0	UD	36.385
53	0	UD	0	UD	0	UD	36.996
54	0	UD	1.095	36.829	0	UD	34.882
55	0	UD	0.155	37.038	0.006	39.204	38.5
56	0	UD	0.031	36.774	0	UD	39.836

Abbreviations: UD – undetermined

Table S5. Friedman Test results obtained from cats with FIP, with the test statistics (**A**) and the Dunn test with Bonferroni adjustment (**B**).

A. Friedman Test to compare the median ranks across multiple assays (RdRP gRNA and mRNA, RdRP mRNA, and E mRNA)

Assay	Mean Rank
RdRP gRNA and mRNA	2.12
RdRP mRNA	2.68
E mRNA	1.20

Test Statistics of Friedman Test	
N	46
Chi-Square	54.663
df	2
Asymptotic Significance	<0.001

B. Friedman Test results with Dunn test and Bonferroni adjustment.

Comparison	Z value	P value adjusted
RdRp gRNA and mRNA vs. RdRp mRNA	-0.8344215	1
RdRp gRNA and mRNA vs. E mRNA	4.3444764	0.000042
RdRp mRNA vs. E mRNA	5.1788979	0.0000007

Table S6. Copy number ratios of the RdRp gRNA and mRNA, RdRp mRNA, E mRNA assay (A) and Intraclass Correlation Coefficient (ICC) of FIP assay Ratios (B).

A. Copy number ratios of the three assays in cats with FIP

Case No	RdRp gRNA and mRNA	RdRP mRNA	E mRNA	RdRP RNA/ RdRp mRNA	RdRP RNA/ E mRNA	RdRp mRNA/ E mRNA
1	0	0	0.005		0	0
2	516.353	1285.454	0.27	0.402	1915.141	4767.722
3	574.387	1284.264	0.848	0.447	677.386	1514.557
4	102.45	132.498	0.792	0.773	129.326	167.257
5	928.643	1131.756	19.591	0.821	47.4	57.768
6	396.031	1112.902	3.561	0.356	111.217	312.535
7	4821.176	1786.531	24.037	2.699	200.572	74.324
8	15.088	27.976	0.001	0.539	42145.67	78146.117
9	0	0.084	0	0		
10	1.857	2.329	0.006	0.8	334.92	418.547
11	3217.579	6630.427	25.572	0.485	125.824	259.285
12	553.827	1444.683	16.702	0.383	33.159	86.496
13	120.682	147.842	90.481	0.816	1.334	1.634
14	0	0.045	0	0		
15	57.539	77.67	236.118	0.741	0.244	0.329
16	126.478	91.843	0.951	1.377	132.977	96.562
17	0	0	0			
18	0.109	0.06	0	1.814		
19	80.48	76.248	0.248	1.056	324.849	307.768
20	28.016	44.736	0.172	0.626	163.011	260.298
21	24.826	68.201	0.123	0.364	201.52	553.597
22	1340.76	2611.608	3.464	0.513	387.1	754.014
23	665.594	1832.123	105.829	0.363	6.289	17.312
24	33.447	65.968	0.424	0.507	78.9	155.615
25	1016.185	1175.98	0.61	0.864	1664.766	1926.551
26	1366.592	2988.876	0.515	0.457	2652.747	5801.829
27	300.404	348.931	2.217	0.861	135.507	157.396
28	399.253	521.234	0.045	0.766	8935.83	11665.927
29	1165.651	664.161	13.708	1.755	85.036	48.452
30	272.655	311.973	1.154	0.874	236.37	270.455
31	534.911	385.036	196.164	1.389	2.727	1.963
32	900.464	1505.415	515.074	0.598	1.748	2.923
33	1.552	2.298	0.006	0.676	245.87	363.923
34	143.442	119.146	0.218	1.204	658.036	546.579
35	1095.653	2641.684	5.212	0.415	210.212	506.834
36	0	0.113	0.003	0	0	39.498
37	0	0.038	0	0		
38	4.228	7553.689	74.375	0.001	0.0568	101.563

39	0.364	0.666	0	0.548		
40	0.521	2.157	0	0.242		
41	0	0.022	0	0		
42	45.708	35.676	0.047	1.281	973.341	759.71
43	110.87	605.314	1.795	0.183	61.784	337.321
44	33.197	6.38	0.057	5.203	579.139	111.309
45	1290.175	423.958	45.716	3.043	28.222	9.274
46	97.251	73.631	0.106	1.321	917.428	694.609

B. Intraclass Correlation Coefficient (ICC) of copy number ratios of the three assays in cats with FIP

Case Processing Summary	Of the 46 cases included into the study, 37 (80.4%) were valid for analysis, and 9 (19.6%) were excluded due to missing data.
Reliability Statistics	Evaluation of the internal consistency among the three assays resulted in a Cronbach's Alpha of 0.683, indicating moderate internal consistency among the measurements and suggesting that the assays measure similar components related to viral load.
Intraclass Correlation Coefficient (ICC)	Single Measures ICC: 0.418 (95% CI: [0.217, 0.613]). Statistically significant ($F(36, 72) = 3.157$, $p < 0.001$), indicating poor to moderate agreement among individual measurements across the different assays.
	Average Measures ICC: 0.683 (95% CI: [0.453, 0.826]). Statistically significant ($F(36, 72) = 3.157$, $p < 0.001$), suggesting moderate agreement when considering the average of the measurements across assays.
Conclusion	The results indicate a notable degree of consistency of viral load ratios among the different assays for cats with FIP. The moderate ICC values suggest that while the assays may provide different absolute values, they yield comparable relative measurements across the sampled population.

Table S7. Mann-Whitney U test group comparison of cats with FIP and FCoV-infected cats without FIP.

Assay	Mann-Whitney U	Wilcoxon W	Z	Asymptotic Significance (2-tailed)	effect size according to Cohen (1992)
RdRp gRNA and mRNA	44.500	99.500	-4.015	<.001	r≈−0.537
RdRp mRNA	42.000	97.000	-4.028	<.001	r≈−0.538
E mRNA	56.000	111.000	-3.766	<.001	r≈−0.503

Table S8. Spearman's rho correlation analysis, illustrating the correlation between the three assays (RdRp gRNA and mRNA, RdRp mRNA and E mRNA) measured for all cats, and for cats with FIP and FCoV-infected cats without FIP separately.

**Variable**	**RdRp gRNA and mRNA**	**RdRp mRNA**	**E mRNA**
**All cats**			
RdRp gRNA and mRNA	1	0.926**	0.853**
RdRp mRNA	0.926**	1	0.878**
E mRNA	0.853**	0.878**	1
**Cats with FIP**			
RdRp gRNA and mRNA	1	0.894**	0.722**
RdRp mRNA	0.894**	1	0.817**
E mRNA	0.722**	0.817**	1
**FCoV-infected cats without FIP**			
RdRp gRNA and mRNA	1	0.588	0.745*
RdRp mRNA	0.588	1	0.702*
E mRNA	0.745*	0.702*	1

* Correlation is significant at the 0.05 level (2-tailed), ** correlation is significant at the 0.01 level (2-tailed)

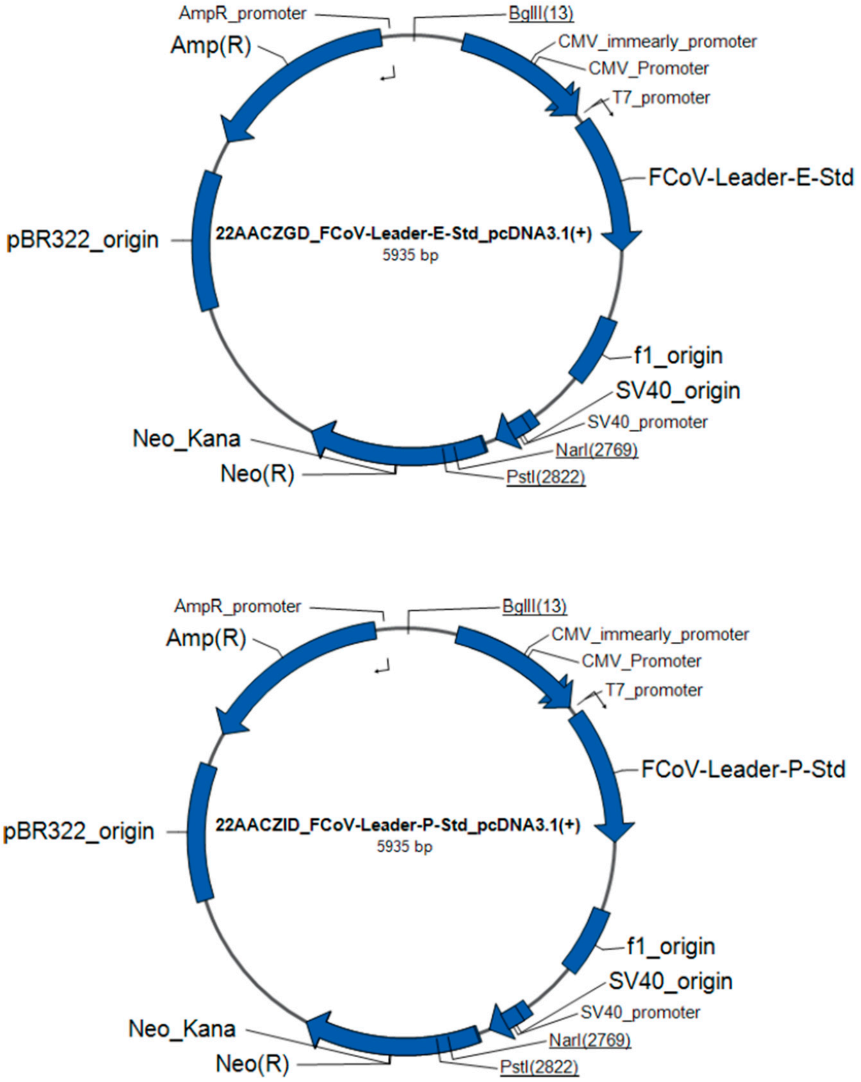

Figure S1. Plasmid maps of the synthetic plasmids with synthetic envelope (above) and polymerase gene (below) from GeneArt Gene Synthesis services (Thermo Fisher Scientific). AmpR (Ampicillin Resistance Gene): Provides resistance to ampicillin for bacterial selection. AmpR promoter: Drives the expression of the AmpR gene. pBR322 origin: A replication origin derived from pBR322 plasmid, enabling plasmid replication in bacterial cells. NeoR/KanR (Neomycin/Kanamycin Resistance Gene): Confers resistance to neomycin and kanamycin for selection in mammalian cells. CMV promoter: A cytomegalovirus immediate early promoter for high-level expression in mammalian cells. T7 promoter: Allows in vitro transcription using T7 RNA polymerase. SV40 origin and promoter: For replication in mammalian cells and transcriptional activity, respectively. F1 origin: Origin for single-stranded DNA production. Restriction sites: Include BglII (position 13), NarI (position 2769), and PstI (position 2822) for cloning and validation.

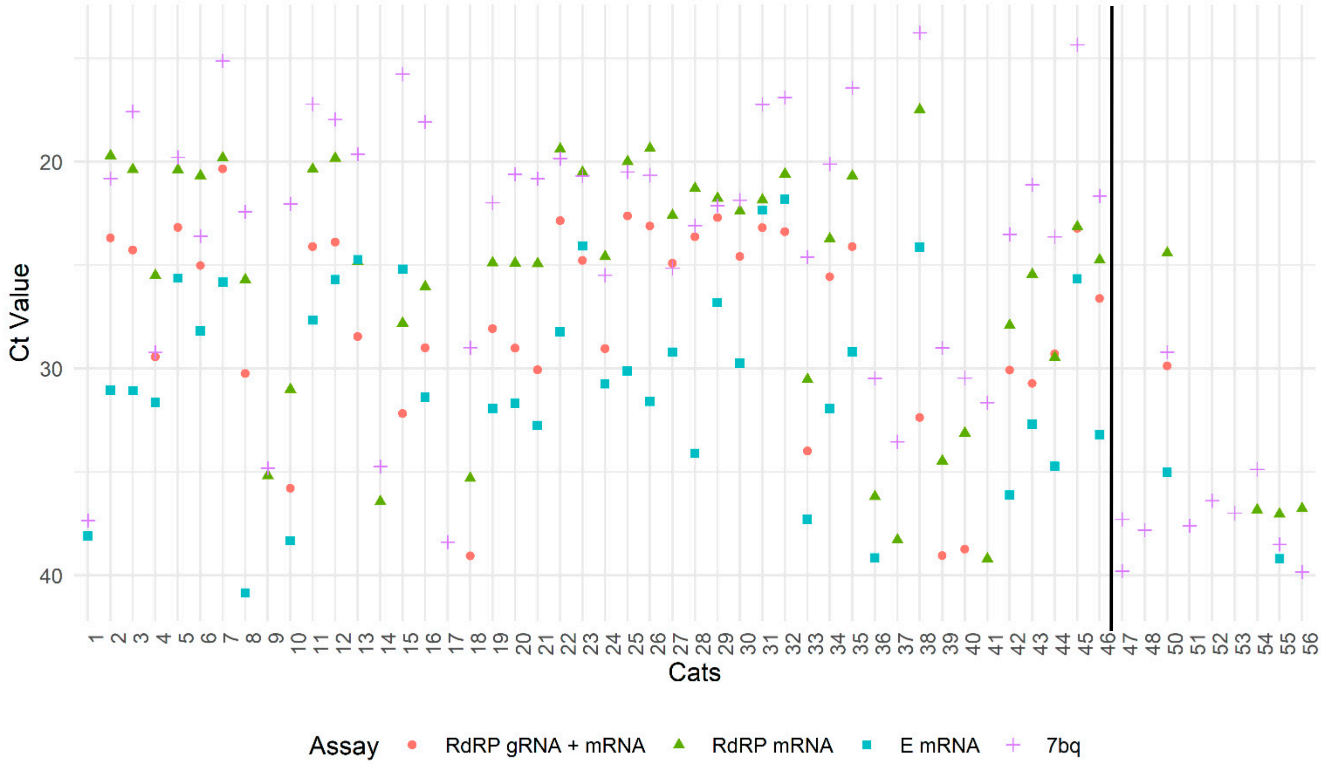

Figure S2. Plot of the Ct values of RdRp gRNA and mRNA, RdRp mRNA, E mRNA, and 7b RT-qPCR in individual cats. Case numbers 1-46 represent the cats with FIP, and 47-56 represent the FCoV-infected cats without FIP.
